# DETECTing Merkel Cell Polyomavirus in Merkel Tumors

**DOI:** 10.3389/fmolb.2020.00010

**Published:** 2020-02-04

**Authors:** Reety Arora, Komal Gupta, Anjali Vijaykumar, Sudhir Krishna

**Affiliations:** ^1^National Centre for Biological Sciences, Tata Institute of Fundamental Research (TIFR), Bangalore, India; ^2^Department of Biology, Indian Institute of Science Education and Research, Pune, India

**Keywords:** Merkel cell carcinoma, Merkel cell polyomavirus (MCPyV), DETECTR, Merkel tumors, molecular diagnosis, CRISPR, CRISPR diagnosis, Cas12a

## Abstract

Merkel cell carcinoma (MCC) is a rare, aggressive skin cancer caused either by Merkel cell polyomavirus (MCV) T antigen expression, post-integration (~80% cases), or by UV-mediated DNA damage. Interestingly, overall survival of MCV-positive Merkel cell carcinoma patients is better, making this differential information of significant diagnostic and prognostic value. Also, MCV provides a direct target for therapy in MCC patients. Currently, the methods used for diagnosis of MCV in tumors are often discordant and unreliable. Here we used a guided molecular scissors based–DNA Endonuclease Targeted CRISPR Trans Reporter (DETECTR) technique to develop an *in vitro* molecular diagnostic tool for MCV-positive MCC. DETECTR couples recombinase polymerase based amplification of target MCV DNA with Cas12a mediated detection. CRISPR diagnostics couple specific detection followed by cutting of the pathogenic DNA by the Cas enzyme–gRNA complex, with non-specific cutting of ssDNA that provides a measurable visual cue. To detect MCV DNA in MCC, we designed Cas12a gRNAs targeting the MCV DNA and tested their targeting efficiency, and sensitivity using a fluorophore quencher labeled reporter assay. We show that MCV DETECTR system can detect MCV integrated in Merkel tumor rapidly, specifically and with femto-molar sensitivity. Our study is a preliminary, proof-of-principle analysis showing the use of CRISPR for MCV diagnosis. Further validation in human tumor samples is needed for its clinical use in the near future. This new system is promising and we hope it can be coupled with immunohistochemical studies to diagnose the viral status of MCC in clinics soon.

## Introduction

Merkel Cell Carcinoma (MCC) is a rare and aggressive neuroendocrine skin cancer (Becker et al., 2017). MCC is associated with old age, excessive UV exposure and weak immune system (Engels et al., 2002; Harms et al., 2018). It is either caused by Merkel cell polyomavirus (MCV) (Feng et al., 2008) or long term Ultraviolet (UV) exposure (Harms et al., 2015; Goh et al., 2016; Starrett et al., 2017).

MCV is the latest addition to the group of human oncoviruses and the only known human oncovirus in the polyomavirus family (Feng et al., 2008). The virus has a double stranded DNA genome, encoding for early and late region genes. Early region genes encode for Small and Large Tumor antigens (sT and LT, respectively) and late region genes encode for the viral proteins (VP) (Feng et al., 2008; Harms et al., 2018).

In virus positive cancers MCV-dependent carcinogenesis requires two events. First, the viral genome gets integrated into the host cell genome, followed by LT getting truncation mutations in its DNA binding and helicase domains to make the virus replication incompetent (Shuda et al., 2008). The expressed T antigens then lead to oncogenesis by altering several pathways and are required for proliferation and survival of MCC cells (Houben et al., 2010, 2012; Shuda et al., 2014). A majority of MCC cases have been found to be MCV positive (~80%) (Feng et al., 2008; Shuda et al., 2009; Becker et al., 2017; Harms et al., 2018).

Both viral positive and viral negative Merkel tumors show identical phenotypes, immunohistochemical (IHC) staining patterns and occur at similar sun-exposed locations on the human body (Hodgson, 2005; Arora et al., 2012, 2019; DeCaprio, 2017; Moore and Chang, 2017; Moshiri et al., 2017; Harms et al., 2018). A pathologist is unable to distinguish between the two types of tumors without the aid of Next-Gen-Sequencing (Goh et al., 2016; Harms et al., 2016). Studies determining the prognostic role of MCV status have had contradictory results, showing either no difference or worsened prognosis of virus negative MCC (Schrama et al., 2011; Hall et al., 2012; Moshiri et al., 2017; Harms et al., 2018). In a recent and the largest study thus far, Moshiri et al. showed that out of 282 MCC tumors, 20% were MCV negative using multimodal qPCR and immunohistochemistry analysis (Moshiri et al., 2017). Interestingly, the study indicated that although both often have aggressive and fatal disease courses, as compared to MCV negative MCC patients, MCV positive MCC patients had significantly better progression-free survival, MCC-specific survival, and overall survival from MCC. Hence identifying the viral status is not only diagnostically (as the cancer cause) important, but important for prognostic predictability as well.

Besides diagnosis and prognosis, the knowledge of MCV integration and T antigen expression in MCC serves are a promising piece of information for MCC treatment as well. The viral DNA sequences and proteins serve as significant external targets that can be exploited for directed therapy for MCV positive cases.

Currently, most commonly used techniques for detecting MCV in MCC tumors include amplifying MCV DNA from DNA isolated from MCC tumors using PCR or immunohistochemistry (IHC) staining for MCV LT using monoclonal antibodies (Guastafierro et al., 2013; Becker et al., 2017; DeCaprio, 2017; Moshiri et al., 2017; Harms et al., 2018). However, these techniques are not the most concordant and reliable. For instance, the Moshiri study using 282 MCC samples used three techniques, IHC with CM2B4 and Ab3 and PCR, for identifying the viral status (Moshiri et al., 2017). However, only 167 / 282 cases showed same viral status in all the three techniques whereas of 282 total cases, 199 cases were positive by qPCR, 205 by CM2B4-IHC, and 254 by Ab3-IHC. Besides, these techniques are usually tedious and required trained personnel to perform them. Thus, there is a need for developing an accurate, sensitive and quick system for distinguishing viral negative vs. viral positive MCC cases (Bhatia et al., 2010; Leroux-Kozal et al., 2015; Brummer et al., 2016; Eid et al., 2017; Harms et al., 2018). In this study we developed a CRISPR/Cas12a based *in vitro* molecular diagnostic system for detecting MCV.

CRISPR (Clustered Regularly Interspaced Short Palindromic Repeats)/Cas system is a genome editing technology derived from the bacterial immune system (Barrangou et al., 2007). CRISPR uses a guide RNA (gRNA) molecule that targets a Cas endonuclease to a specific genomic site using sequence homology and PAM (Protospacer adjacent motif) recognition. Upon target binding Cas protein induces a double strand break in the target (Hsu et al., 2014; Swarts et al., 2017; Gupta et al., 2019). Cas12a (Also known as Cpf1) is a type V CRISPR protein having various properties distinct from Cas9. Cas12a enzymes recognize a T nucleotide–rich protospacer-adjacent motif (PAM), catalyze their own guide CRISPR RNA (crRNA) maturation, and generate a PAM-distal dsDNA break with staggered 5′ and 3′ ends (Barrangou et al., 2007; Dong et al., 2016; Murugan et al., 2017). Interestingly, unlike Cas9, after dsDNA target binding the Cas12a enzyme leads to indiscriminate trans ssDNA cleavage activity (Chen et al., 2018). Chen et al. used this property to develop DNA endonuclease-targeted CRISPR trans reporter (DETECTR) system which can rapidly and specifically detect target HPV DNA (Chen et al., 2018).

We adapted the DETECTR system to detect the presence of MCV DNA integration in the Merkel cell tumor genome. Briefly, we assemble a reaction mix containing AsCas12a, MCV specific gRNA, test DNA, and fluorophore-quencher (FQ) ssDNA substrate in a tube. In the presence of MCV DNA, Cas12a binds and cleaves the cis target DNA followed by trans cleavage of fluorescently labeled ssDNA. This Cas12a mediated DNase activity leads to fluorescence based detection of MCV.

Here we show that when combined with Recombinase Polymerase based amplification of target DNA, DETECTR system can identify MCV with femtomolar sensitivity. DETECTR can detect MCV in MCV positive MCC cells efficiently and specifically. We tested our system on MCC cell lines as a preliminary validation of the system. Testing of this assay on clinical human MCC samples is still required and will add further credibility to our study. Thus, we hope that this MCV DNA detecting system can then be coupled with histopathological and immunohistochemical studies to diagnose the viral status in MCC and help guide clinicians in the near future.

## Materials and Methods

### sgRNA Design

Ten AsCpf1 gRNAs were designed to target the Non-coding Control Region (NCCR), small and large Tumor (sT and LT, respectively) antigen of MCV. Two CRISPR gRNA design tools: Benchling (https://benchling.com/pub/cpf1) and RGEN (www.rgenome.net/cas-designer) were used. MKL-1 (Genbank Accession #: FJ173815.1) and MS-1 (Genbank Accession #: JX045709.1) MCV sequences were used as target DNA sequences. gRNAs with highest specificity score and with lowest possible off targets were selected. MCV gRNA target region sequence conservation was analyzed using NCBI BLAST (https://blast.ncbi.nlm.nih.gov/Blast.cgi).

### Synthesis of sgRNAs Using IVT

For *in vitro* transcription (IVT), two oligonucleotides were designed as DNA templates, per gRNA. Forward oligonucleotide for AsCas12a gRNA synthesis consisted of the T7 promoter (TAATACGACTCACTATAGG) followed by AsCas12a sgRNA scaffold and 20-nucleotide long guide RNA. Three G's were added after T7 promoter sequence for efficient T7 transcription.

Target substrate for AsCas12a NCCR gRNA1 *in vitro* cleavage (pam/TARGET):

5′ GGCCTCTCTCTTTTtttc CAGAGGCCTCGGAGGCTAGGAGCCCCAAGCCTCTG 3′

crRNA oligo for *in vitro* transcription (**T7** / ADDED G/*SCAFFOLD*/GUIDE):

Forward oligo:

**TAATACGACTCACTATA**GGG*TAATTTCTACTCTTGTAGATCAGAGGCCTCG*GAGGCTAGG

Reverse oligo:

CCTAGCCTCCGAGGCCTCTG*ATCTACAAGAGTAGAAATTA*CCC**TATAGTGAGTCGTATTA**

Forward and reverse oligonucleotides were annealed by heating to 95°C for 5 min and slow cooling on bench top. sgRNAs were synthesized using the MEGAShortScript^TM^ Kit (# AM1354, Invitrogen) following the manufacturer's protocol with 2 μg annealed oligonucleotides template, overnight at 37°C. The RNA was treated with 1 μl of DNase TURBO for 15 min followed by purification using the MEGAclear Transcription Clean-Up Kit (# AM1908, Invitrogen) (see Supplementary Table 1 for all oligonucleotides and primer details).

### *In vitro* Cleavage Assay

*In vitro* cleavage reaction was performed at 37°C in cleavage buffer consisting of 20 mM HEPES (pH 7.5), 150 mM KCl, 10 mM MgCl_2_, 1% glycerol and 0.5 mM DTT. Thirty nanometers AsCas12a (#1081068, IDT) and 36 nM gRNA were pre-assembled in the cleavage buffer at 37°C for 10 min. 18.5 nM dsDNA target was added to the reaction (20 μl). PCR amplicons of NCCR, sT, and LT regions from plasmid RAZ2 (Addgene #114382) were used as dsDNA target templates. The PCR amplicon was purified using Ampure beads (#A63881, Beckman Coulter) as per manufacturer's protocol. For M13 cleavage assays, 30 nM AsCas12 and 36 nM gRNA were preassembled at 37°C for 10 min in cleavage buffer. Forty nanometers dsDNA activator and 10 nM single stranded M13mp18 phage (#N4040S, NEB) were added to initiate the reaction (30 μl). The reaction was incubated at 37°C for 60 min. The IVC reactions were stopped by treatment with 2 μl of Proteinase K (10 mg/ml) at 55°C for 10 min. 1 × gel loading dye was added to the reactions and samples were run on 2% agarose gels (# RM273, HIMEDIA).

### Fluorophore Quencher (FQ)-Labeled Reporter Assays

200 nM AsCas12a, 250 nM gRNA and 18.5 nM dsDNA target template were pre-assembled in a 5 μl reaction for 30 min at 37°C. Reaction was initiated by diluting these complexes to AsCas12a: gRNA: dsDNA template to 50 nM: 62.5 nM: 4.6 nM in 1x Binding buffer (20 mM Tris-HCl, pH 7.5, 100 mM KCl, 5 mM MgCl2, 1 mM DTT, 5% glycerol, 50 μg ml^−1^ heparin) and 50 nM custom ssDNA FQ reporter substrate (IDT). The custom ssDNA reporter substrate consisted of 5′ 6-FAM™, 5 nucleotides long oligo (TTATT) and 3′ Iowa Black® FQ. The 20 μl reactions were incubated at 37°C for 1 h in a 384 well-microplate format. The fluorescence was measured using Tecan (Infinite M Plex) (Excitation: 485 nm, Emission: 535 nm). Experiments were repeated independently three times with two technical repeats each time. The graphs were drawn using GraphPad Prism and statistical significance was calculated using one way ANOVA.

### Cell Culture

HEK293T, U20S, and BJhTERT cell lines were obtained from ATCC and grown in Dulbecco's Modified Eagle Medium (DMEM) supplemented with 10% Fetal Bovine Serum (FBS), 1X Penicillin/Streptomycin (Pen Strep, catalog # 15140122). Merkel Cell Carcinoma cell line MKL-1 was obtained from ECACC (# 09111801) and MS-1, MCC26, and WaGa cells were kind gifts from Dr. James Decaprio's laboratory at Dana Farber Cancer Institute, Boston. MCC cell lines were cultured in Roswell Park Memorial Institute (RPMI) Media with 10% FBS and 1X Penicillin/Streptomycin. The cells were grown at 37°C with 5% CO_2_.

### Genomic DNA Isolation

10^6^ cells were harvested for genomic DNA extraction. Cells were lysed in Tris EDTA buffer and 200 μg/mL Proteinase K at 55°C for 1 h. After Proteinase K was heat inactivated, 1 μl of this lysed sample was used for DETECTR experiments.

### Recombinase Polymerase Reaction (RPA)

For DETECTR experiments, genomic DNA was first amplified using recombinase polymerase reaction (RPA). The RPA reaction mix was prepared by adding 0.48 μM forward primer and reverse primers, 29.5 μl primer Free Rehydration buffer, 1 μl of extracted genomic DNA and water to 13.2 μl (Total volume 47.5 μl). The reaction mix was added to one TwistAmp^®^ Basic (#TABAS03KIT, TwistDx) reaction tube. 2.5 μl of 280 mM Magnesium Acetate (MgOAc) was added and mixed to start the reaction. The reaction was incubated at 37°C for 20 min.

### DETECTR Assay for Genomic DNA

DETECTR uses RPA reaction to amplify target DNA followed by Cas12a based detection. Eighteen microliter of the RPA reaction mix was assembled with 2 μl of 50 nM AsCas12a, 62.5 nM gRNA and 50 nM custom ssDNA FQ substrate. Reaction was incubated at 37°C for 1 h and fluorescence was measured using Tecan Infinite Pro 200.

For investigating DETECTR's sensitivity, a titration experiment was performed. NCCR, sT and LT target region was amplified from plasmid RAZ2 (Addgene #114382) and used as dsDNA target. This dsDNA was diluted to varying concentrations ranging from 10^−8^ to 10^−15^ M. Fluorophore quencher (FQ)-labeled reporter assay was performed as described previously using these targets with or without RPA. A variation of this mix, without any DNA target, but with gRNA and Cas12a enzyme and excess buffer was used as a “no target control” for normalization of emitted fluorescence and fold change calculation.

## Results

### gRNAs Were Designed to Target All MCV Variants

**Figure 1:**
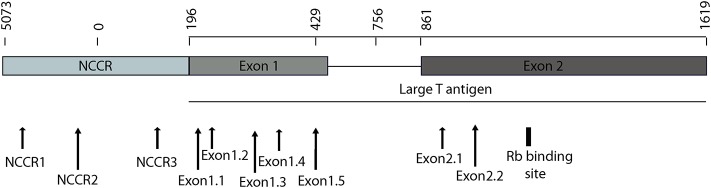
MCV NCCR and early region map showing gRNA locations. The map above shows the location of 10 gRNAs (AsCas12a CRISPR based) that we designed and the specific sites on the MCV genome that they target.

To detect the presence of integrated MCV DNA in MCC cells, we designed 10 guide RNAs compatible with AsCas12a. Three gRNAs are complementary to the Non-Coding Control Region (NCCR), 5 gRNAs target Exon 1 of T antigen and the remaining 2 gRNAs target Exon 2. We used MKL-1 MCV sequence (Genbank Accession #: FJ173815.1), till the conserved Retinoblastoma (Rb) binding domain region to design these gRNAs (Figure 1 and Table 1). All gRNAs designed targeted conserved regions across MCV subtypes known so far (Schowalter et al., 2010; Matsushita et al., 2014a; Buck et al., 2016). Using Clustal Omega Multiple Sequence Alignment we checked 52 MCV sequences (from NCBI) across all gRNA-targeting regions.

**Table 1:** AsCas12a_gRNAs against Merkel cell polyomavirus.

	**gRNA name**	**Strand**	**Sequence**	**PAM**	MCV genome location^*^	**Benchling score^#^**	**RGEN score^#^**
1	NCCR1	Minus	AACAAGGGAGGCCCGGAGGC	TTTC	5156-5137	98.01	66.3
2	NCCR2	Minus	CTGGAGAGGCGGAGTTTGAC	TTTC	5239-5220	98.24	74.2
3	NCCR3	Minus	CAGAGGCCTCGGAGGCTAGG	TTTC	26-7	95.67	69.1
4	Exon1.1	Minus	GGACTAAATCCATCTTGTCT	TTTA	208-189	92.81	60.1
5	Exon1.2	Plus	GAGATTGCTCCTAATTGTTA	TTTA	247-266	93.40	62.4
6	Exon1.3	Plus	GTCCTAAATAGGAAAGAAAGAGA	TTTA	205-227	79.10	73.9
7	Exon1.4	Minus	CCCCTTTATCAGGGTGATGCTTT	TTTC	334-312	93.34	79.4
8	Exon1.5	Minus	CTCCAAAGGGTGTTCAATTCCAT	TTTG	374-352	95.56	75.2
9	Exon2.1	Plus	CCATCTAGGTTGACGAGGCC	TTTC	853-872	99.32	75.0
10	Exon2.2	Minus	TGGATCTTGAGTTGGTCCCG	TTTC	962-981	98.04	69.3

* *MCV Genome Location is based on MKL-1 genome (GenBank: FJ173815.1)*.

# *Benching and RGEN are softwares whose scores are out of 100*.

### Screening and Validation of gRNAs Targeting MCV Genome

**Figure 2:**
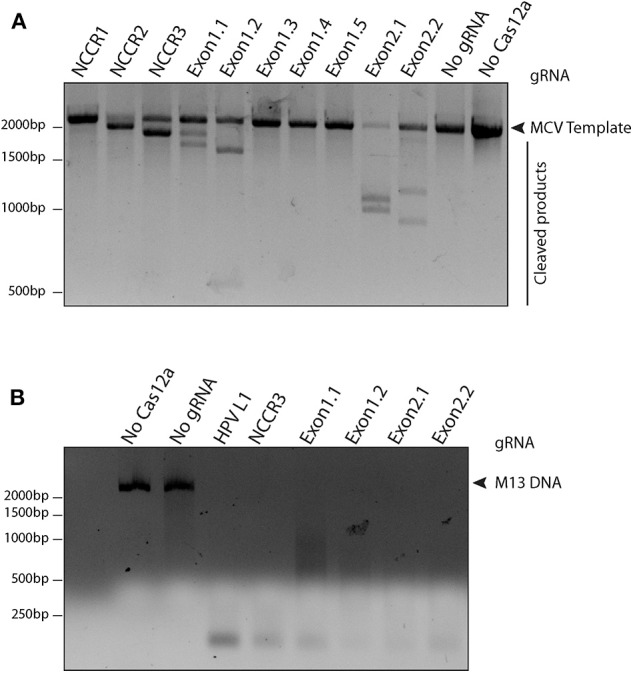
Screening gRNAs for efficient cleavage. **(A)** *in vitro* cleavage (IVC) assay for all gRNAs. An MCV template of 2,193 bp spanning the NCCR region to the RB-binding site of T antigen was used for the assay (shown by the arrow). Of the 10 gRNAs tested, six showed efficient cleavage that resulted in visible chopped bands on a 2% agarose gel. **(B)** Non-specific trans ssDNA shredding activity of AsCas12a-gRNA. M13 DNA phage reporter was subjected to MCV gRNA, complementary ssDNA target (cis-activator) and AsCas12a. All five gRNAs, namely NCCR3, Exon1.1, Exon1.2, Exon2.1, and Exon2.2 resulted in indiscriminate shredding of single-stranded M13 DNA.

To access the cleavage efficiency of these gRNAs, we performed *in vitro* cleavage assays. We used the 2,193 bp long NCCR and T antigen region (derived from MS-1) as template/ DNA substrate for our assays. Six out of 10 gRNAs efficiently cleaved the target substrate as shown by the cleaved bands (Figure 2A).

Next, to validate the non-specific trans ssDNA shredding activity of AsCas12a, we used a non-complementary, circular, single stranded M13 DNA phage reporter. In the presence of MCV gRNA and complementary ssDNA target (cis–activator), AsCas12a led to indiscriminate shredding of single stranded M13 phage (Figure 2B). Thus, 5 MCV gRNAs: NCCR3, Exon 1.1, Exon 1.2, Exon 2.1, and Exon 2.2, were selected for further studies for detecting MCV.

### Detection of MCV via Fluorescence Measurement

**Figure 3:**
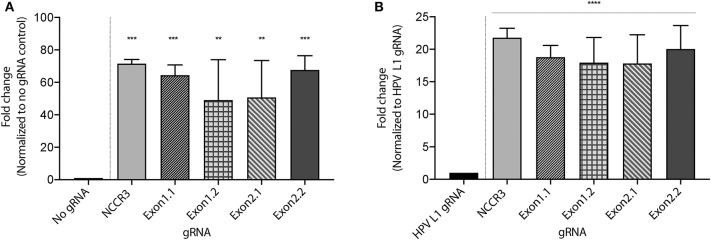
MCV detection via fluorescence measurement. A Fluorophore-Quencher (FQ) labeled reporter assay was used to test the gRNA-AsCas12a combinations. MCV gRNA, AsCas12a and complementary dsDNA cis target were assembled and subjected to a custom ssDNA FQ reporter (excitation 485 nm, emission 535 nm). All five gRNAs showed significant fold change in emitted fluorescence (y axis) as compared to No gRNA control [~48-fold and above, **(A)**] and HPV L1 gRNA control [~18-fold and above, **(B)**]. Error bars represent SD for three independent experiments. One-way ANOVA with Dunnett test was performed for statistical analysis. (Adjusted *p*-values: p_NCCR3_ = 0.0003, p_Exon1.1_ = 0.0007, p_Exon1.2_ = 0.0065, p_Exon2.1_ = 0.005, and p_Exon2.2_ = 0.0005, ^**^p < 0.01, ^***^p < 0.001 for **(A)** and for **(B)** ^****^p < 0.0001).

To develop a MCV DNA detection system, we used a fluorophore quencher (FQ)–labeled reporter assay. We assembled AsCas12a with its MCV gRNA and a complementary dsDNA cis target. We introduced a non-specific ssDNA FQ reporter to this reaction. In the presence of gRNA targeting MCV DNA and complementary target MCV DNA, AsCas12a cleaved the ssDNA FQ reporter as observed through the emitted fluorescence. We found that all the five selected gRNAs showed significant fold change in emitted fluorescence as compared to no gRNA reaction and HPV L1 targeting gRNA as controls (Figure 3 and Supplementary Figure 1). HPV L1 targeting gRNA and matching template were used as a positive control for the assays (Supplementary Figure 2; Chen et al., 2018).

**Figure 4:**
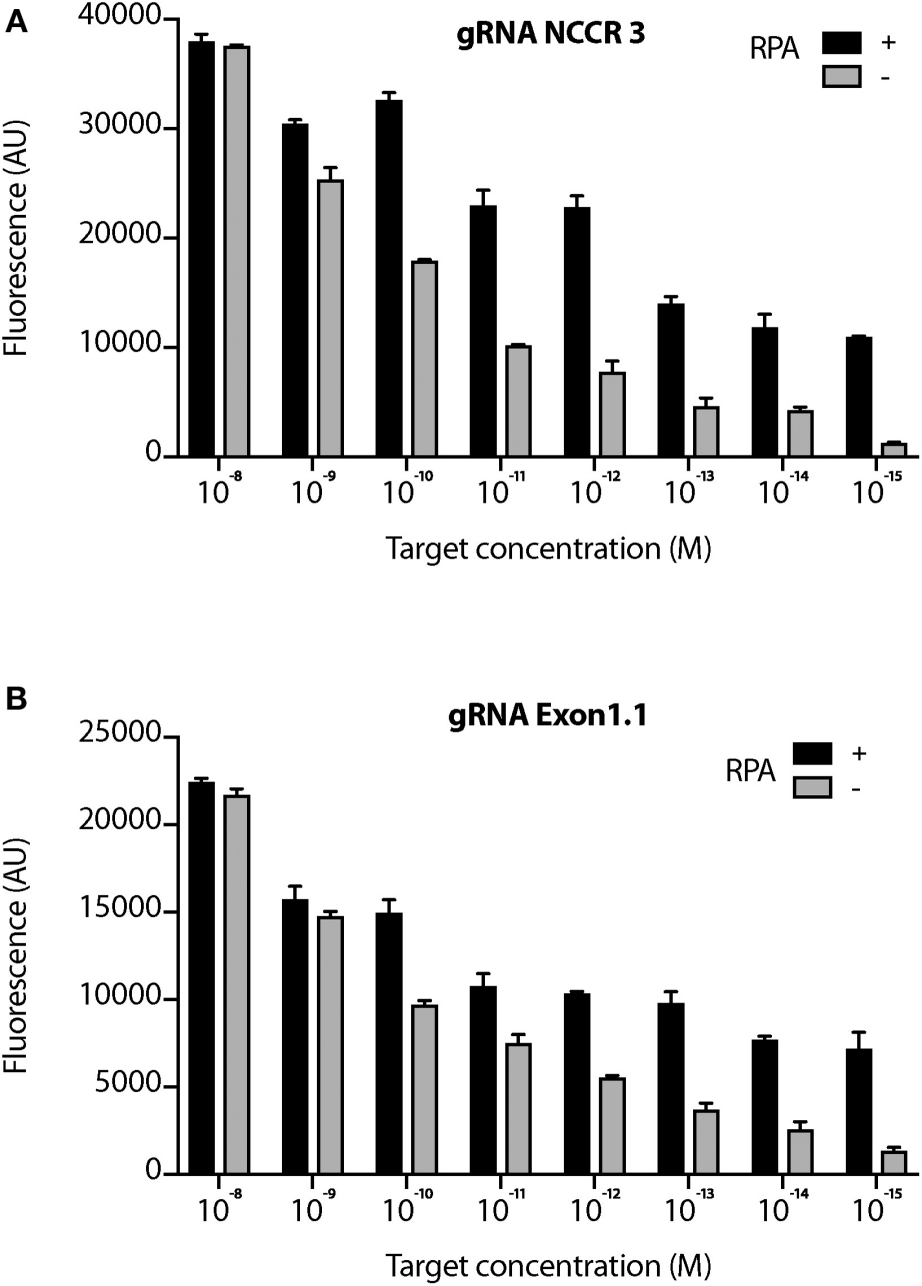
Sensitivity of MCV gRNA to detect low copies of MCV. The Cas12a based detection of MCV was performed in the presence and absence of RPA (Recombinant Polymerase Amplification) with varying concentrations (range- 10^−15^ to 10^−8^ M target concentration) for both **(A)** gRNA NCCR3 and **(B)** gRNA Exon1.1. y-axis represents background subtracted emitted fluorescence. Error bars represent SD for three independent experiments.

We proceeded further with 2 gRNAs: NCCR 3 and Exon 1.1 after validating them against “No Target control” (Supplementary Figure 3). The DETECTR from Prof. Doudna's group, couples Recombinase Polymerase with Cas12a based DNA detection (Chen et al., 2018). To investigate the sensitivity of our MCV DETECTR system, we used a plasmid containing the MS-1 NCCR and T antigen region with varying concentrations (range- 10^−15^-10^−8^ M). We performed Cas12a based detection using these targets with and without Recombinase Polymerase Amplification. Our results show that the MCV DETECTR (RPA + Cas12a) system we created can efficiently detect MCV DNA up to femtomolar concentration (Figure 4).

### DETECTR Can Diagnose MCV in MCC Genomic DNA

Next, we investigated whether DETECTR can specifically and efficiently detect MCV in MCV positive MCC cell's genomic DNA. We extracted genomic DNA from various cultured human cells infected with MCV (MKL-1, MS-1, WaGa) or without MCV (MCC26, BJhTERT, U2OS). Whereas, MKL-1, MS-1, WaGa and MCC26 are all Merkel cell carcinoma cell lines, BJhTERT is an immortalized fibroblast cell line and U2OS is an osteosarcoma cell line.

**Figure 5:**
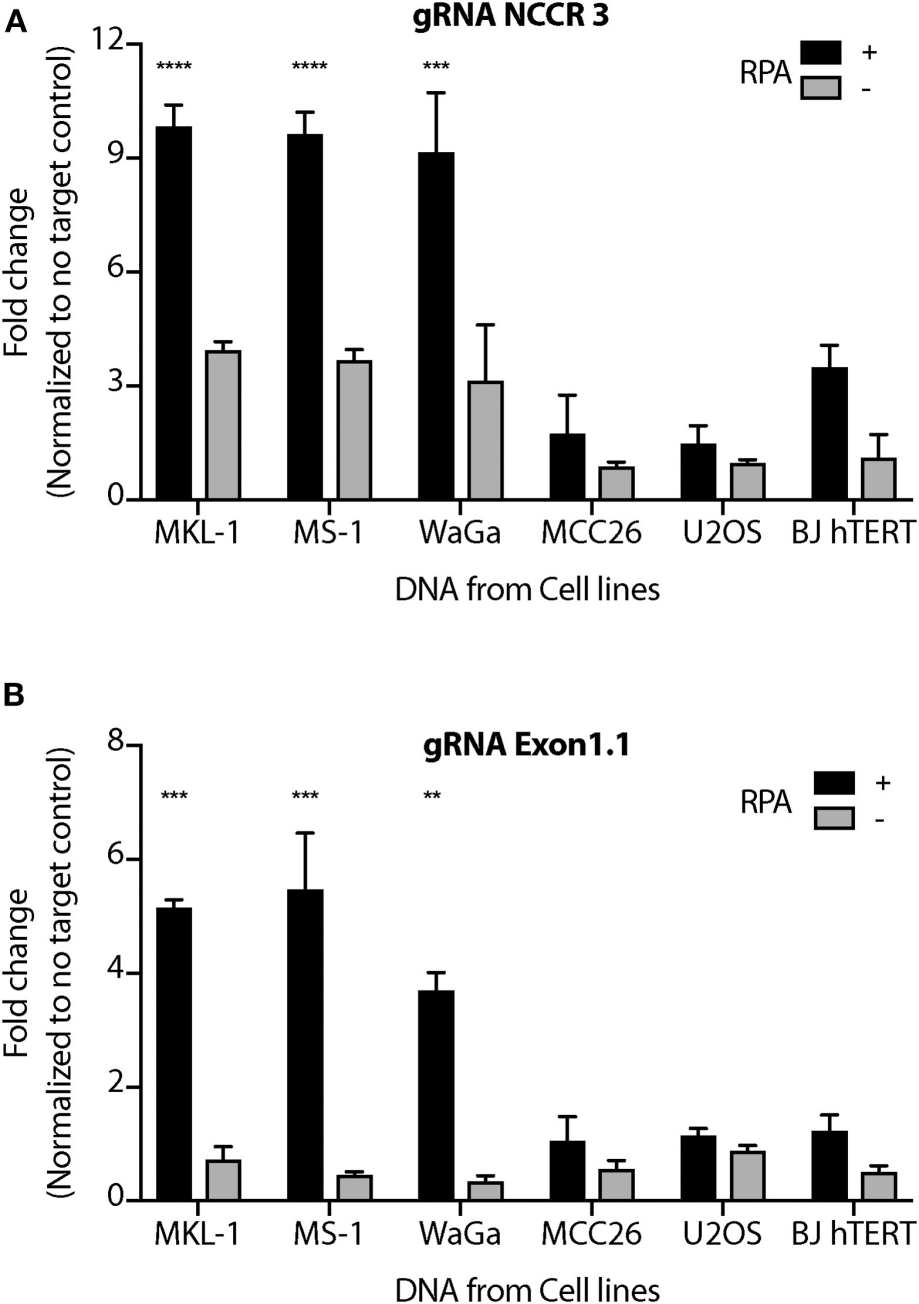
MCV DETECTR in action. Genomic DNA from MCV positive MCC cell lines MKL-1, MS-1, WaGa; MCV negative MCC cell lines MCC26; Osteosarcoma cell lines U2OS and immortalized Fibroblasts BJhTERT were extracted and subjected to the MCV DETECTR assay. With the use of RPA, MCV was detected significantly in all MCV MCC positive cell lines for both **(A)** gRNA NCCR and **(B)** gRNA Exon 1.1. Fluorescence was normalized to no-target control and fold change of emitted fluorescence plotted. Error bars represent SD for two independent experiments One-way ANOVA with Dunnett test was performed for statistical analysis (^**^p < 0.01, ^***^p < 0.001, ^****^p < 0.0001).

**Figure 6:**
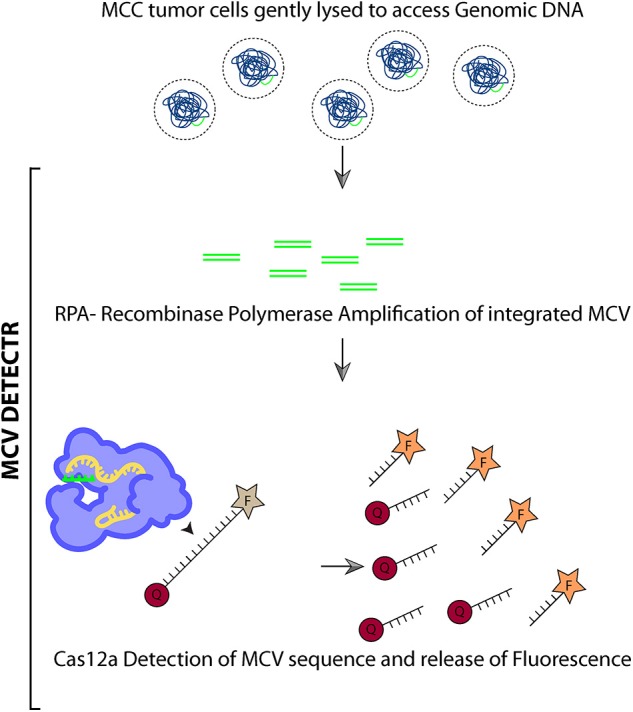
Schematic summarizing MCV DETECTR.

We performed MCV detection in these samples using AsCas12a DETECTR (RPA + Cas12a). We found that although only Cas12a-gRNA complex was not sensitive enough to identify MCV, DETECTR unambiguously detected MCV in MKL-1, MS-1, and WaGa cells (Figure 5 and Supplementary Figure 4). Also, there was no significant signal in the presence of non-complimentary DNA from MCV negative cells. Thus, we show that the MCV DETECTR can be potentially used as a specific and efficient platform for diagnosing MCV in MCC tumors (Figure 6).

## Discussion

Currently, the methods used for the experimental detection or diagnosis of MCV in tumors include immunohistochemistry, PCR, RNA, or DNA *in situ* hybridization and next-generation sequencing (NGS) (Shuda et al., 2009; Duncavage et al., 2011; Haugg et al., 2011; Rodig et al., 2012; Matsushita et al., 2014b; Wang et al., 2017; Harms et al., 2018). All of these assays vary considerably in terms of sensitivity and specificity for MCV detection in tumors. Immunohistochemical measurement of T antigen protein expression is the commonest approach for MCV detection and the antibody used for the same is usually CM2B4 (88% sensitivity, 94% specificity; Moshiri et al., 2017). Recently Moshiri et al. introduced a more efficient multimodal approach combining PCR and immunohistochemistry for detection of viral T antigens (Moshiri et al., 2017).

Although sensitive, these methods do have their drawbacks. For examples, CM2B4 often gives non-specific staining in both tonsillar and lymphoid tissues (Leroux-Kozal et al., 2015; Moshiri et al., 2017). Other antibodies that detect MCV T antigens, such as Ab3 and Ab5 have been reported, however none have been used as frequently as CM2B4 for detecting viral proteins in the clinic (Cheng et al., 2013; Harms et al., 2018; Arora et al., 2019).

PCR is another easy and common method used for MCV detection by many laboratories. In comparison with the multimodal approach, PCR-based amplification within the second exon of LT had 83% sensitivity and 81% specificity (Moshiri et al., 2017). Quantitative PCR (qPCR) extends this analysis and allows for estimation of number of integrated MCPyV copies per host cell genome (Katano et al., 2009; Sihto et al., 2009). MCPyV copy number estimates have been reported to range from <1 copy per 100 cells to thousands of copies per cell (Shuda et al., 2009; Rodig et al., 2012).

Technical factors, including inefficient PCR amplification owing to mutations in the integrated MCPyV genome, primer incompatibilities, low purity of tumor samples or the detection of infectious wild-type MCPyV in the adjacent non-malignant skin often confound the results for such tests (Eid et al., 2017; Wang et al., 2017). Besides, qPCR does not allow for visual confirmation that positive results are associated with tumor cells; hence, background MCV presence cannot be excluded in MCC tumors with low signal (Wang et al., 2017).

RNA *in situ* hybridization and NGS are other newer approaches being investigated for MCPyV detection. RNA *in situ* hybridization might provide PCR level sensitivity in conjunction with visual correlation with tissue morphology and the exclusion of background infection (Wang et al., 2017). NGS, on the other hand is effective in detecting MCPyV sequences, including tumor-specific truncating mutations and viral integration sites (Duncavage et al., 2011; Starrett et al., 2017). However, the time, expense and expertise required for these methods currently make these approaches impractical for many diagnostic and research laboratories.

The “cut and paste” function of CRISPR is popular and what the system is often associated with, after a guide RNA finds a target DNA sequence, the Cas nuclease makes a double-stranded cut and one can paste a sequence of choice at that site. The newer Cas12 and Cas13 have enabled researchers to use the “search” function of CRISPR in diagnostics (Liu et al., 2019). Platforms such as DETECTR by Jennifer Doudna's group (Chen et al., 2018) and SHERLOCK (Gootenberg et al., 2017, 2018) by Feng Zhang's group have leveraged this search functionality to get sensitive and specific diagnostic results for HPV and Dengue virus, respectively. Further studies to use CRISPR for Zika virus, Lassa fever, and even Ebola are underway (Chertow, 2018; Pan and Kraschel, 2018; Hadidi, 2019; Pickar-Oliver and Gersbach, 2019). The startups called Mammoth Biosciences and SHERLOCK Biosciences are such endeavors for providing better diagnostics for disease.

These non-editing applications of CRISPR have brought to light a precise, easy and efficient detection system for disease and here we have extended Dr. Doudna's group's studies to the tumor virus Merkel cell polyomavirus.

Our work offers a novel diagnostic method for MCV detection (Figure 6). Our MCV DETECTR approach is accurate, efficient, easy to use and rapid (30 min). It has a clear readout (fluorescence), specific and sensitive to femto-molar levels. The simplicity of this method, lack of complex sample preparation and no requirement for expensive equipment makes it superior to others currently being used. This CRISPR trans reporter system for DNA detection has opened a new applications door for CRISPR based technologies with rapid and specific detection of Merkel cell polyomavirus in patient samples. Additionally, combining this approach with immunohistochemistry and/or PCR will substantially improve the sensitivities of detection of MCV.

In our study, we tested the CRISPR system we developed for MCV on MCC cell lines for validation. Although robust and promising, further testing on clinical human MCC samples is needed and will add more value to the use of this system and further assist in taking it to clinical use as a diagnostic tool.

Thereby, our system is a simple platform for molecularly diagnosing whether a tumor is virus positive or negative. This information is not only an important causal indicator for Merkel tumors but also a powerful prognosis predictor. We hope that it will soon be incorporated into clinical practice and positively affect MCC studies.

## Data Availability Statement

All datasets generated for this study are included in the article/Supplementary Material.

## Author Contributions

RA and KG performed the experiments and wrote the manuscript. AV provided important reagents and performed the experiments. AV and SK provided intellectual support and valuable discussions. All authors listed have made a substantial, direct and intellectual contribution to the work, and approved it for publication.

### Conflict of Interest

The authors declare that the research was conducted in the absence of any commercial or financial relationships that could be construed as a potential conflict of interest.
